# Supplementary material for: Strain design optimization using reinforcement learning
Source: PLoS Comput Biol. 2022 Jun 3;18(6):e1010177. doi: 10.1371/journal.pcbi.1010177 (PMC9200333; doi:10.1371/journal.pcbi.1010177)
Supplement: Attachment — Submitted filename: response.pdf [file pcbi.1010177.s001.pdf]

We thank the reviewers for reading the manuscript and for their positive and constructive feedback. Based on that, we have significantly revised the manuscript. In particular, for experimental validation, we have added new experimental results on L-tryptophan production in *Saccharomyces cerevisiae* that rely on a combinatorial strain library from Zhang et al. 2020 (https://www.nature.com/articles/s41467-020-17910-1). In addition, we have substantially revised the k-ecoli457 simulation studies.

Reviewer #1: The authors recognise that strain optimization is a combinatorial search problem, and that RL is a useful approach in such cases (provided the data – real or simulated - are available). Of course in the hands of DeepMind RL has had considerable success e.g. in learning to play Go, and so on. They (wisely) choose a model-free approach, not least since there are no directly predictive relations between the observables (i) concentration and (ii) flux (both are variables).

I set out thinking that this would be very exciting, but TBH it promised rather more than it delivered. I think the gushing tone of the abstract needs to be toned down to recognise the limitations more clearly: (i) only three somewhat unexciting fluxes were measured, (ii) it required FORTY iterations, which is a huge number for an experimental program, and (iii) many of the explanations were close to non-existent. It probably reflects the mathematical strengths of the first author, but these cannot come – in PlosBiol at least – at the expense of explaining what the findings actually meant. In consequence, it failed to convince me that I might seek to adopt their strategy, which is what I would wish it to have done. (Or alternatively that it was a nice idea but not in fact worth adopting in this way, a point nicely made in Harford T: How to Make the World Add Up: ten rules for thinking differently about numbers. London: Bridge Stree Press, 2020.)

We thank the reviewer for the critical evaluation of our manuscript. We agree that for any experimental setting 40 iterations is too many for practical relevance. However, the focus of the paper was to evaluate the strain design algorithms' capabilities. Therefore, we chose to report the result at the point where all the algorithms are closer to convergence to the optimum. We have now added a new Figure (Figure 3) that shows the development of the response variable through the iterations. From there it can be established that ca. 15 iterations suffices for MARL to get close to the optimum in all three products. We have added new results optimization of strain design for L-tryptophan overproduction in yeast *S. cerevisiae* (Figure 5), where around 15 iterations suffices to get average response to within 90% of the optimum. We have also modified the abstract accordingly and added a paragraph in the discussion about the complexity of the algorithm w.r.t the number of iterations required.

Abstract: “We demonstrate the method’s capabilities in comprehensive experiments using the genome-scale kinetic model of E. coli, k-ecoli457, as a surrogate for in vivo cell behavior”. These were simulations, not experiments. Call them so. Comprehensive is a claim, not a fact.

Thank you for the precise corrections suggestion, we substituted “experiments“ with “simulation”.

I am not qualified other than to take the maths on pp 3-4 on trust. The background of the authors suggests this is reasonable.
The multi-agent aspect is nice as it recognises that this is effectively how experiments are performed in a DBTL cycle.
The authors might care to contrast their approach with BOED as in
Foster A, Jankowiak M, Bingham E, Horsfall P, Teh YW, Rainforth T, Goodman N: Variational Bayesian Optimal Experimental Design. arXiv 2019:1903.05480v05483.
Vanlier J, Tiemann CA, Hilbers PAJ, van Riel NAW: A Bayesian approach to targeted experiment design. Bioinformatics 2012; 28:1136-1142.

Thank you for the suggestion. However, we already include the BO-GP approach as a comparison method that relies on Bayesian optimization. A border comparison of methods could be an interesting future work direction.

Fig 2. Numbers in heat map unreadable. Also try to choose non red-green for those who are colour blind. https://colorbrewer2.org/#type=diverging&scheme=BrBG&n=3 gives suggestions.
“As we observe in this figure, the algoritmh [sic] tunes the enzyme levels in generally smooth fashion while the larger changes correspond to replacing the worst agent with another”. Maybe so, but I cannot see this and so it needs a much better explanation. Tell me what I am supposed to look at and what I am supposed to draw from it.

We thank the referee for this comment. We concluded that the figure was too complicated and did not adequately serve its purpose to illustrate the algorithm’s behavior. We replaced the figure with one that visualizes the enzyme levels of a single agent through the iterations. The colors and the font size of the numbers in the heatmap have been improved.

I don’t understand the units in Figs 2 and 3 – you can’t have more moles out than went in…

We thank the reviewer very much for the careful work and for pointing this out. The unrealistic values were independent of our method but resulted from the kinetic model used as a proxy for experiments not converging in all cases using the convergence tolerance proposed by the model developers. We found the kinetic model convergence slow with more stringent tolerance but we were able to successfully use the proposed tolerance for optimizing designs for maximizing product exchange flux * growth instead of yield. This modification for demonstrating the performance of our method, however, was only necessary due to the nature of the kinetic model and not our RL method. The target used was also highly relevant as it does not lead to designs severely compromising the cell viability.

Yield improvement p8. 40 iteration is not that realistic for real DBTL programs. It would be good to show the time course of the median per iteration.

Thank you for the interesting suggestion. We have added a new figure (Figure 3) to the manuscript, in which we show the percentage of improvement of MARL with respect to BO-GP and RAND in the evolution of the iterations, across the three investigated problems. After changing the target to optimizing designs that maximize the response variable (product exchange flux * growth) we could also show that within 15 iterations, designs substantially improving the target could be found. With this few iterations needed our method has high practical relevance to be integrated to the experimental DBTL cycle.

Fig 3. Why is succinate different from ethanol and acetate?
In the new set of experiments that we carried out the response is considered “product exchange flux * growth” rather than “yield”, which succinate seems to have similar behavior to the other two products.

Table 2. Strain stability measure. Needs far more explanation – what am I supposed to infer? Also I doubt that the second DP has much meaning, or even the first…

We do not completely understand this remark (what is DP short-hand for?). However, the intent is to study how sensitive the final strain design obtained by the algorithm is to small variations in the obtained enzyme levels, i.e. how much will the response values (here: growth-coupled production) change if the actual enzyme levels are different from the planned ones (which we would expect to happen). The results show that the MARL algorithm tends to find designs that give more statistically stable results than the RAND approach in all of the studied three cases and better results than BO-GP in two of the three cases.

Fig 4. Again you tell us what you did but not what we are supposed to make of the results.

Figure 4 depicts how the performance of the MARL algorithm changes upon different levels of noise arising from different sources (measurement errors, biological variability, inaccuracy of implementing enzyme level changes).
Since the k-ecoli457 model is deterministic, it lacks all of these sources of variability and, consequently, might make the algorithm's performance look unrealistically good compared to the real-life situation. Here we have added different levels of noise to the inputs and outputs of the algorithm to simulate the sources of variability. Figure 4 shows that overall, the optimum response level obtained by the algorithm decreases proportionally to the amount of noise added. Also we notice that noise in the response variable (here: product exchange flux * growth) is the most damaging form of noise while noise in the actions (i.e. inaccuracy of implementing planned enzyme levels) is the least damaging form of noise.

"The enzyme levels can be quantified using targeted mass spectrometry based proteomics approaches found useful in optimizing production". I doubt it. Transcriptomics typically provide a much better surrogate (Machado D, Herrgård M: Systematic evaluation of methods for integration of transcriptomic data into constraint-based models of metabolism. PLoS Comput Biol 2014; 10:e1003580).

Actually, transcriptomics does not provide a good surrogate for metabolic function in cells. Machado and Herrgård, appreciatively referred to by the reviewer, found and state that: "Also, it is observed that for many conditions, the predictions obtained by simple flux balance analysis using growth maximization and parsimony criteria are as good or better than those obtained using methods that incorporate transcriptomic data." Thus, optimizing transcriptomics as the desired fluxes is not reasonable as it cannot be expected to lead into desired changes in the metabolic function. However, our RL method is equally suited for designing strategies adjusting protein and gene expression levels for optimizing a target. Due to the complexity of the post-transcriptional regulation, the learning could be expected to be slower and not even the gene expression levels can be set exactly using experimental methods. Exploiting the targeted proteomics is likely to lead to most efficient strain optimization through a DBTL cycle.

Reviewer #2: *This is a good paper, the proposed reinforcement learning (RL) method is novel to the field of strain engineering, well explained and sound. The benchmark with other methods is also fair and clear. The introduction of noise is fully relevant especially*

where dealing with strain engineering. Yet, I find the method a bit limited in scope. First, one needs a strain ODE kinetics models and very few are available. Secondly, the method is applied only to a predefined set of enzymes and that information is not always available. Finally, to experimentally implement the method one needs to regulate the expression levels of enzymes (via promoters, RBSs..) and this is not a straightforward task where model simulations always agree with experimental results.

Unfortunately there seems to be a misunderstanding of our RL method. It is model free, no ODE kinetics of the metabolic enzymes of the organism are needed. In this work we use the kinetic model of E. coli only as a proxy for laboratory experiments. It is not part of the RL method. When integrated to an experimental DBTL cycle, the RL method receives rewards from the experimental characterization of the engineered strains. We have clarified this in the revised version of the manuscript. We have also included another performance demonstration case, optimizing strain designs for maximizing L-tryptophan production with *S. cerevisiae*. In this case, we can evaluate the performance of our RL method using experimental data from strains engineered by Zhang et al. 2020 (https://www.nature.com/articles/s41467-020-17910-1).

I would advise the authors to add in the discussion the current limitations of their method and discus if and how a similar method could be developed and used with genome scale models (GEMs) and steady state dynamics as there are plenty of such models, methods and results in the literature. The authors could also comment and if and how the actions could be simplified to propose set of genes to be knocked out. Note that in that latter case and when using GEMs, results could be compared with MILP solvers like OptKnock.

It is indeed true that modifying metabolic enzyme levels is not a straightforward task. Metabolic gene deletions would be simpler to implement in wetlab. However, with metabolic gene deletions only not much flux redistribution towards improving production can be done. Most of the competing pathways are essential for growth and sufficient activity has to be maintained for cell viability, growth and stability. Therefore, the metabolic enzyme level changes are essential for developing industrially feasible microbial production strains. Fortunately, the latest synthetic biology tools offer already for many organisms promoter libraries for a range of different expression values (e.g. Lee et al. 2015, https://pubs.acs.org/doi/full/10.1021/sb500366v) and core promoters that are applicable across organisms and that can be tuned for different activities (Rantasalo et al. 2018, doi: 10.1093/nar/gky558; Deng et al. 2021, DOI: 10.1186/s12934-021-01691-3). In addition, approaches such as synthetic degrons (e.g. Chasson et al. 2019, doi: 10.1038/s41467-019-09974-5) are available for tuning the enzyme levels by altering the enzyme degradation.

Identification of the target enzymes whose levels should be tuned for optimizing production has previously been successfully identified using a stoichiometric model Zhang et al. 2020 (https://www.nature.com/articles/s41467-020-17910-1). We believe this is a strong approach relying on mass conservation and thermodynamic constraints of metabolism, and the number of enzymes to be included in the RL framework can be varied. Optimizing the actual enzyme levels for maximizing production calls for a data driven approach like our RL method due to the complexity of cellular regulation with the ultimate limits of mass conservation and thermodynamics.

Minor comments:

Page 2: In addition to references 6-8 which make use of machine learning but not RL there are few papers explicitly making use of RL in the context of bioproduction and it is worth mentioning these //doi.org/10.1016/j.jprocont.2018.07.013 , //doi.org/10.1021/acssynbio.9b00447, //doi.org/10.1016/j.compchemeng.2019.106649 , //doi.org/10.1371/journal.pcbi.1007783 .

Thank you for your suggestion, we added the mentioned studies to the references.

Figure 1. It would be wise to define the symbols used in the caption. Some can be found in the text but not all. Would also be wise to indicate where in the flow chart the policy is learned and used.

The caption is modified to include the symbols. Also we have added about policy in the figure 1, where it is learned and used.

Page 4. The ML engine to build the policy is MMR (SVM based), could the author motivate this choice as other methods could have been used?

MMR allows to learn vector outputs with internal structure, therefore the potential interdependence between the output components can be expressed. MMR is the simplest known approach to implement these kinds of regression problems. The alternatives, e.g. StructuredSVM, and the Input Output Kernel Regression, have significantly higher computational complexity, thus their use in an iterative, Reinforcement Learning framework could be too expensive. The computational complexity of solving the optimization problem realizing an MMR based learner is the same as the complexity of the SVM applied to binary classification.

Page 5. The authors make use of a mixed centralized and decentralized training and defined groups where RL is carried out. Although within a group, exploration seems to

be favored, I do not see the RL exploitation vs. exploration strategy being used nor discussed. Could the authors comment on that?

The MARL algorithm attempts to strike a balance between exploration and exploitation. Within the group, the agents' initial actions are towards the maximum predicted reward i.e. fully in exploitation mode. The within-group perturbation of the actions increases the degree of exploration while the replacement of the worst agent slightly encourages exploitation.

Table 1. The target reaction needs to be defined.
Target reaction is defined as the corresponding reaction to the product of interest, which for each product is defined in table 1.

Figure. 2. The process is iterated 40 times but the Figure shows only 13 rows, what are they? Would also be good to state that the numbers and colors correspond to enzyme level.

The rows corresponded to iterations where the agents found an improved design. We have now replaced the figure with a simplified one, where the enzyme levels are visualized for a single agent. We have updated the figure caption to explain the figure elements.

Figure 4. The Figure shows yield decrement when noise is introduced separately to action, yield and state, has any test been carried out where noise was introduced simultaneously on the 3 elements?

Yes indeed it would be interesting to include the case where noise has been introduced to the three elements simultaneously as this case resembles the noise which could be present in a laboratory testing environment. We have included it in this Figure 4.

In addition there are few typos which could be cleared with a spell checker.

Thank you for pointing this out. We have corrected the typos in the revised version of the manuscript.

Reviewer #3: This is an excellent paper outlining an important new approach to strain design optimization. I have two main areas of feedback:

Thank you for your encouraging comments.

1) The specific settings/decisions for tuning the MARL parameters are not justified. How was it decided for example the number of iterations and trajectories? Would slightly different options yield improved performance? Would one set of parameters be universally best for any problem, or would users of this method need to re-evaluate this for each new context? This should be explained.

We have added to the experiments a plot (Figure 3) in which we show how the proposed algorithm works, throughout the iterations.
Regarding the number of agents, we had tried different setups (higher number of agents in each group, higher number of groups, ec.) and we did not see any significant difference, however, lower number of agents and groups could deteriorate the results. In the new dataset that we have included in the last section (study on the experimental dataset provided in https://doi.org/10.1038/s41467-020-17910-1) the same setup and parameters worked fine too, which we mentioned in the text.

2) Substantial editing is needed to improve the clarity of the manuscript. For example: on line 239 "algorithm" is spelled wrong and the word "a" is missing. The sentence starting on line 254 is grammatically incorrect.

Thank you for pointing this out. We have corrected the grammatical mistakes and the typos.
