# Supplementary material for: Strain design optimization using reinforcement learning
Source: PLoS Comput Biol. 2022 Jun 3;18(6):e1010177. doi: 10.1371/journal.pcbi.1010177 (PMC9200333; doi:10.1371/journal.pcbi.1010177)
Supplement: Attachment — Submitted filename: response_r2.pdf [file pcbi.1010177.s002.pdf]

We thank the reviewers for reading the manuscript and for their positive feedback.

Reviewer #2: "I would advise the authors to add in the discussion the current limitations of their method and discus if and how a similar method could be developed and used with genome scale models (GEMs) and steady state dynamics as there are plenty of such models, methods and results in the literature. The authors could also comment and if and how the actions could be simplified to propose set of genes to be knocked out. Note that in that latter case and when using GEMs, results could be compared with MILP solvers like OptKnock."

The development of out RL method does not require any mechanistic model to be known. We used the kinetic model only as a replacement of laboratory experiments to demonstrate our MARL method. In the latter case, we demonstrated MARL with real experimental data and in this case no mechanistic model of metabolism was used. **We have now clarified this also in the discussion part of our manuscript.** While for the current method we don't assume any mechanistic model of metabolism to be known, the target enzymes to MARL could be identified with the help of genome-scale metabolic models if no suitable experimental information is available. Being limited to optimizing the levels of the given target enzymes is also the current limitation of MARL method. **These points have now been added to the discussion part of our manuscript.** Our MARL method could also be further developed to benefit from the stoichiometry of the species metabolism if it is known. We could for instance predict beneficial gene deletions using genome-scale metabolic models before initializing MARL optimization or use fluxes predicted for modified strains using genome-scale metabolic model simulations as input for MARL. **We have now included this in the discussion part of our manuscript.** Several algorithms using genome-scale metabolic models for predicting fluxes of mutant strains exist (e.g. MoMA [Segre et al. 2002], ROOM [Shlomi et al. 2005]) that do not assume evolutionarily optimized metabolic status as flux balance analysis does [Varma and Palsson, 1994] but rather aim at maintaining the wild type flux regulation as much as possible. Strain design methods using genome-scale metabolic models (such as OptKnock [Burgard et al. 2003], FSEOF [Choi et al. 2010]) rely on these simulation algorithms, either FBA or the ones not assuming optimal growth, for predicting mutant strain flux status. The predictions of flux states following from metabolic gene deletions using genome-scale metabolic models are quite good and have been shown successful for supporting strain improvement and the metabolic gene deletions would be simple to implement in wetlab. However, with metabolic gene deletions only not much flux redistribution towards improving

production can be done. Most of the competing pathways are essential for growth and sufficient activity has to be maintained for cell viability, growth and stability. Therefore, the metabolic enzyme level changes are essential for developing industrially feasible microbial production strains. **This point has now been clarified in the discussion section of our manuscript.**

Most importantly, however, the regulation of metabolism involves high biological complexity beyond the stoichiometry and the kinetic constraints that we are able to mechanistically model. Our RL method is aimed at being able to learn that limitedly known complex regulation in addition to the stoichiometric and kinetic constraints, and in this way to make a leap in the model-guided strain design.
